# LY450139 Inhibited Ti-Particle-Induced Bone Dissolution via Suppressing Notch and NF-κB Signaling Pathways

**DOI:** 10.1007/s00223-022-00980-2

**Published:** 2022-05-19

**Authors:** Jijian Gao, Peng Wu, Yingjun Chi, Hongyu Xu, Yong Zhao, Nanyan Song, Yuanqing Mao

**Affiliations:** 1 Department of Orthopaedic Surgery, Shengzhou People’s Hospital (the First Affiliated Hospital of Zhejiang University Shengzhou Branch), Shaoxing, 312000 Zhejiang China; 2 Shanghai Key Laboratory of Orthopaedic Implants, Department of Orthopaedic Surgery, Shanghai Ninth People’s Hospital, Shanghai Jiao Tong University School of Medicine, Shanghai, China; 3 Department of Orthopaedic Surgery, Huating First People’s Hospital, Pingliang, 744000 Gansu China

**Keywords:** LY450139, Notch, Osteoclast, NF-κB, Osteolysis

## Abstract

Aseptic loosening of the prosthesis caused by wear-particle-induced osteolysis is a long-term complication and one of the most common reasons for the failure of joint implants. The primary cause of aseptic loosening of the prosthesis is overactive bone resorption caused by wear-particle-activated osteoclasts in both direct and indirect ways. Therefore, drugs that can inhibit differentiation and bone resorption of osteoclasts need investigation as a potential therapeutic strategy to prevent and treat peri-prosthetic osteolysis and thereby prolong the service life of the prosthesis. This study has verified the potential inhibitory effect of LY450139 on inflammatory osteolysis induced by titanium particles in a mice skull model. In addition, we found that LY450139 inhibited receptor activator of NF-κB ligand (RANKL)-induced osteoclastogenesis, bone resorption, and podosomal actin belt formation in a dose-dependent manner without evidence of cytotoxicity in vitro. In addition, LY450139 significantly decreased the expression of osteoclast-specific markers, including TRAP, CTSK, V-ATPase d2, CTR, DC-STAMP, NFATc1, and the downstream target gene Hes1 in Notch signaling pathway. Further investigation of the molecular mechanism demonstrated that LY450139 inhibited the formation of osteoclasts via inhibition of the NF-κB and Notch signaling pathways. In summary, LY450139 inhibited the formation of RANKL-mediated osteoclasts via NF-κB and Notch signaling and inhibited Ti particle-induced inflammatory osteolysis in vivo. LY450139 is a potential targeted drug for the treatment of peri-prosthetic osteolysis and other osteolytic disease associated with overactive osteoclasts.

## Introduction

In the field of joint surgery, more and more total hip replacements are performed each year to treat various joint-related diseases, such as femoral neck fractures in the elderly, ischemic aseptic necrosis of the femoral head, degenerative osteoarthritis, rheumatoid arthritis, and malignant bone tumor of the hip joint [1, 2]. Although the patients were satisfied with the treatment effect after the operation, an unfortunate shortcoming of prostheses is their limited lifespan [3]. Previous studies have shown that during the wear process, titanium particles, as the most representative worn sub-micrometer particle debris causes secretion of pro-inflammatory cytokines by macrophages, production of pro-resorptive cytokines by osteoblasts and fibroblasts, stimulation of osteoclastogenesis, induction of osteolysis, and ultimately loosening of the implant [4, 5]. Regrettably, total hip replacement revision surgery tends to be conducted to solve this intractable problem. A specific, persistent chronic inflammatory response is induced by implant particles at the implant-bone interface [6], recruiting macrophages, fibroblasts, neutrophils, lymphocytes, osteoblasts, osteoclasts, and pro-inflammatory secretion, including bioactive amines (histamine and serotonin), cytokines (IL-1 and TNF-α), and chemokines, all of which contribute to the process of osteolysis [5]. As activated by chronic inflammatory responses, osteoclasts exhibit excessive bone resorption behavior that disrupts the stability of bone formation [7, 8]. Osteoclasts are multinucleated giant cells formed by the fusion of mononuclear macrophages derived from the bone marrow [9]. Early immature proliferative mononuclear phagocytes, known as osteoclast precursors, enter the bloodstream and are recruited to bone tissues in the absorbent state under the influence of chemokines, then differentiate into osteoclasts [10, 11]. The formation and function of osteoclasts are controlled by molecular mechanisms involving multiple signaling pathways [12, 13]. The binding of m-CSF to c-FMS maintains the proliferation of osteoclast precursor cells, while binding of RANKL to RANK is necessary for the differentiation, function, and survival of mature osteoclasts [14, 15].

Cells perceive and integrate information from the external environment and transmit information from the outside to the inside of the cell to produce a proper developmental or physiological response. Reports have showed that Notch signaling pathway, an evolutionarily conserved information transmission mechanism, affects the individual cell differentiation of species and regulates many critical biological processes, including growth, development, metabolism, apoptosis, homeostasis, and pathogenesis [16]. During signal transmission, the intracellular portion of protein in Notch pathway is cleaved by the γ‐secretase complex to generate Notch intracellular domain (NICD) [17], as a second messenger, which enters the nucleus to regulate Myc, P21, Hes Family, and CyclinD3 Target genes [18]. Notch pathway can regulate bone and cartilage development and homeostasis maintenance, also plays a role in the development of pathological conditions, such as joint degenerative disease osteoarthritis (OA) and cancers including primary bone cancer and metastatic cancer. Notch signaling pathway is a key regulator of osteoclastogenesis and bone resorption. Previous studies have reported that Notch pathway has stimulating effects on RANKL-induced osteoclast formation and bone destruction. Therefore, inhibiting Notch receptor proteolysis also inhibits Notch pathway, which may inhibit osteoclast generation in turn, with significance for clinical treatment of osteolysis-related diseases.

Semagacestat (LY450139), a γ-secretase inhibitor (GSI), has been reported to play an important role in treating Alzheimer's disease via modulating Notch pathway, whereas, its application in the orthopedics field is rarely reported so far [19, 20]. Thus, to investigate the potential role of Notch pathway in Ti particle-induced peri-prosthetic osteolysis, we used LY450139 to inhibit Notch activity in RANKL-induced osteoclastogenesis and observed the changes in osteoclast formation and function after inhibiting Notch signaling pathway. We also explored the potential mechanisms behind these osteoclast changes to search for putative therapeutic targets in osteolysis.

## Materials and Methods

### Chemicals, Reagents, and Antibodies

The γ‐secretase inhibitor LY450139 was purchased from Selleck (Houston). The alpha-modified minimal essential medium (α-MEM) and fetal bovine serum (FBS) were purchased from Gibco BRL (Gaithersburg, MD, USA). Recombinant Mice M-CSF Protein and RANKL were manufactured by R&D Systems (Minneapolis, MN, USA). A cell counting kit (CCK-8) for cell proliferation and viability was obtained from Dojindo Molecular Technology (Kumamoto, Japan). Tartrate-resistant acid phosphatase (TRAP) stain was supplied from Sigma-Aldrich (St Louis, MO, USA). Specific antibodies against IκBa, p-IκBa, NFκB p65, p-NFκB p65, NICD2, Hes1, NFATc1, β-actin, and fluorochrome conjugated secondary antibodies were purchased from Cell Signaling Technology (Cambridge, MA).

### Titanium Particles

Pure titanium (Ti) particles (> 93% in purity, diameter ranging from 2 to 20 µm and with 90% < 5 μm) were obtained from a commercial source (Alfa Aesar, Haverhill, MA, USA). Ti particles were sterilized by baking at 180 °C for 6 h and washed in 75% ethanol for 48 h for endotoxin removal. Subsequently, the particles were resuspended with sterile phosphate-buffered saline (PBS) at a concentration of 300 mg/mL and deposited at 4 °C until use. The endotoxin level of particle suspension was tested with Limulus assay (Biowhittaker, Walkersville, MD), according to the manufacturer's instructions.

### Ti Particle-Induced Calvaria Osteolysis Mice Model

Ti particle-induced mice calvarial osteolysis model in this study was established as described by the previous study [20]. The use of laboratory animals was approved by the Animal Experimentation Ethics Committee of Shanghai Jiao Tong University Affiliated Ninth People’s Hospital. Twenty-four male C57BL/6 mice (8-week-old) (Shanghai Slac Laboratory Animal Company) were kept under specific pathogen-free conditions and separated into four groups of six mice each. The four groups consisted of the following: the sham group was treated with PBS; the vehicle group was treated with Ti particles in PBS, the low‐dose group was treated with titanium particles and LY450139 (500 μg/kg), and the high‐dose group was treated with titanium particles and LY450139 (2000 μg/kg). The mice were anesthetized by 1% pentobarbital sodium (45 mg/kg) via intraperitoneal injection. An approximately 1-cm incision was made on the skin of the calvaria center area, and the periosteum was stripped laterally along the centerline for exposure of the calvaria. Then, 30 mg of Ti particles were embedded under the periosteum at the mid-line of the calvaria before suture. A sham operation (making a mid-line sagittal incision over the calvaria and suturing with local injecting 100 μL PBS) was taken as the blank control. The wound healed almost completely 3 days after the operation. Then, the low‐dose group (500 μg/kg) and high‐dose group (2000 μg/kg) were injected under the scalp with different drug concentrations, while the other groups were injected with PBS every other day for 14 days. No deaths or complications were observed during the 14 days’ observation. Finally, on the 14th day of injection, the mice were sacrificed and the calvaria of each mice was excised and fixed in 4% paraformaldehyde for micro-computed tomography (µ-CT) and histological analysis.

### Micro-CT Scanning

The fixed calvarias were examined by high-resolution micro-CT scanning (Skyscan 1072; Skyscan, Aartselaar, Belgium). Scanning was performed at an X-ray tube voltage of 80 kV, a current of 80 mA, and an exposure time of 300 ms, respectively, with an isometric resolution of 18 μm. To enable a quantitative analysis of bone mineral density expressed as the concentration of hydroxyapatite, we calibrated micro-CT imaging protocol using Solid hydroxyapatite (HA) calibration phantoms (SCANCO Medical AG, Bruttisellen, Switzerland) with concentrations of 0, 100, 200, 400, and 800 mg HA/cm^3^. Phantoms with a known density of CaHA were employed to calibrate the instrument and generated the proper conversion of linear relationship, then the X-ray attenuation coefficients produced in micro-CT are converted to an equivalent BMD value (mg HA/cm^3^). As previously described, After reconstruction, we chose a square region of interest (ROI; 3 × 3 × 1 mm) around the mid-line suture for further qualitative and quantitative analysis. The parameters were evaluated with CT-Analyser were as follows: bone mineral density (BMD), bone volume/total tissue volume (BV/TV), pore number, and the percentage of porosity (1-BV/TV) according to ASBMR standard guidelines [21].

### Histological Staining

For histological analysis, after micro-CT scanning, calvarias samples were decalcified using 10% disodium ethylenediaminetetraacetic acid (EDTA, pH 7.4) for 4 weeks at 4 °C, then processed for paraffin embedding using standard methods and cut into 6 μm thick sections. Histological sections were stained with hematoxylin and eosin (H&E) and TRAP. Specimen images were examined under a microscope (Leica Microsystems, Wetzlar, Germany). The number of TRAP-positive cells and osteoclast surface per bone surface (OCS/BS %) were quantified using ImageJ software.

### Mice Bone Marrow Macrophages (BMMs) Isolation and Culture

Six‐week‐old C57BL/6 male mice were sacrificed under sterile conditions. BMMs were extracted from the medullary cavities of femurs and tibias and incubated in a 100 mm culture dish with α‐MEM, 10% FBS, and 30 ng/mL M‐CSF in a humidified environment of 5% CO_2_ and 37 °C. The culture medium was changed every 2 days.

### Cell Viability/Cytotoxicity Assay

Cell proliferation and drug toxicity of LY450139 were tested with a CCK-8 kit following the manufacturer’s instructions. BMMs (1 × 10^4^ cells/well) were seeded into 96-well plates in triplicate and cultured in 100 μL α-MEM supplemented with M‐CSF (30 ng/mL) and graded doses of LY450139 (0, 1.25, 2.5, 5, 10, 20, 30 and 40 μM) for 48, 72, 96 h. Next, 10 μL of CCK-8 reagent was added to each well for an additional 2 h of incubation. Finally, absorbance (optical density, OD) was read at 450 nm (reference wavelength 630 nm) using an automated microplate reader.

### RANKL-Induced Osteoclastogenesis and TRAP staining Assay In Vitro

For osteoclast differentiation, BMMs (1 × 10^4^ cells/well) were seeded into a 96-well plate in triplicate and provided with a complete α-MEM medium with M-CSF (30 ng/mL), RANKL (50 ng/mL), and various concentrations of LY450139 (0, 1, 5 or 10 μM). Culture medium was replaced every 2 days until osteoclast formation. The cells were fixed with 4% paraformaldehyde for 20 min and rinsed three times by PBS, then stained with TRAP. TRAP-positive multinucleated cells that contained three or more nuclei were counted as osteoclasts (OCs). The staining results were assessed and quantified using Image J software.

### Podosome Actin Belt Immunofluorescence Staining

Glass slides were placed in a 24-well plate, and BMMs were seeded in triplicate wells of 24‐well plates at a density of 2 × 10^5^ cells per well. When mature osteoclasts appeared in control wells (5–7 days), osteoclasts were fixed with 4% paraformaldehyde for 20 min and permeabilized with 0.1% Triton X-100 (Sigma-Aldrich, St. Louis, Missouri) for 5 min. Cells were then incubated for 30 min in darkness with rhodamine-conjugated phalloidin diluted in PBS containing 1% bovine serum albumin (BSA). After washing (4 × PBS for 5 min), cells were counterstained with 4′, 6-diamidino-2-phenylindole (DAPI) for 5 min. The image was captured using Leica fluorescent microscope and analyzed using Image J software.

### Hydroxyapatite Resorption Pit Assay

Osteoclast function assays were performed by measuring hydroxyapatite resorption in vitro. BMMs (1 × 10^4^ cells/well) were seeded into hydroxyapatite-containing 96-well plates in triplicate and treated with M-CSF (30 ng/mL), RANKL (50 ng/mL), and LY450139 (0, 1, 5, or 10 μM) until mature osteoclasts formed. After gently brushing the cells with sodium hypochlorite solution, the erosion mark of the hydroxyapatite pit surface was visualized and imaged with a BioTek Cytation 3 Cell Imaging Reader (BioTek, Winooski, VT), and five view fields were randomly selected for each pit. The area percentage of bone resorbed was quantified using Image J software.

### RNA Extraction and Quantitative Real-Time PCR Analysis

BMMs (4 × 10^5^ cells/well) were cultured with M-CSF (30 ng/mL) and RANKL (10 ng/mL). Cells were then treated with LY450139 (0, 1, 5, or 10 μM) for 5–6 days. Total mRNA was extracted from the BMMs with TRIzol Reagent (Takara Biotechnology, Otsu, Japan). cDNA was synthesized using the Revert Aid First-strand cDNA with the Prime Script RT Reagent Kit. PCR was conducted with a real-time fluorescent quantitative PCR System. The comparative 2^−ΔΔCT^ method was utilized to quantify the relative expression levels of each gene as previously. GAPDH was a housekeeping gene. The following primer sets were used as previously described (Table 1).

**Table 1:** Primers for real-time PCR

Source	Name	Forward	Reverse
Mice	TRAP	5ʹ- CTGGAGTGCACGATGCCAGCGACA -3ʹ	5ʹ- TCCGTGCTCGGCGATGGACCAGA -3ʹ
	CTSK	5ʹ- CTTCCAATACGTGCAGCAGA -3ʹ	5ʹ- TCTTCAGGGCTTTCTCGTTC -3ʹ
	CTR	5ʹ- TGCAGACAACTCTTGGTTGG -3ʹ	5ʹ- TCGGTTTCTTCTCCTCTGGA -3ʹ
	NFATc1	5ʹ- CCGTTGCTTCCAGAAAATAACA -3ʹ	5ʹ- TGTGGGATGTGAACTCGGAA -3ʹ
	V-ATPase d2	5ʹ- AAGCCTTTGTTTGACGCTGT -3ʹ	5ʹ- TTCGATGCCTCTGTGAGATG -3ʹ
	DC-STAMP	5ʹ- AAAACCCTTGGGCTGTTCTT -3ʹ	5ʹ- TTCGATGCCTCTGTGAGATG -3ʹ
	Hes1	5ʹ-CTGAGGCAAGGATTGGAGTC-3ʹ	5ʹ-GAATGGAGGTAGGTGCGAAG-3ʹ
	GAPDH	5ʹ- GGTGAAGGTCGGTGTGAACG -3ʹ	5ʹ- CTCGCTCCTGGAAGATGGTG -3ʹ

### Western Blotting Analysis

Western blotting analysis was performed as previously described [22]. The cells were seeded into 6-well plates at a density of 4 × 10^5^ cells/well. To assess long-term effects, BMMs were stimulated with M-CSF (30 ng/mL), RANKL (50 ng/mL), with or without LY450139 (10 μM) treatment for 0, 1, 3, or 5 days. To measure short-term effects, cells were pretreated with serum-free α-MEM, with or without LY450139 (10 μM) for 2 h, and then stimulated with RANKL (50 ng/mL) for 0, 5, 10, 20, 30, and 60 min. Cells from each experimental group were lysed in radioimmunoprecipitation assay (RIPA) buffer, and total protein concentrations were determined using a BCA Protein Assay Kit (BioTek). Next, proteins (10 μg/sample) were separated by 10% SDS-PAGE and transferred onto polyvinylidene fluoride (PVDF) membranes. The membranes were blocked with 5% (w/v) non-fat dried skimmed milk powder in wash buffer (Tris-buffered saline/1% Tween-20) for 1 h, then incubated with primary antibodies overnight at a dilution recommended by the suppliers. The membranes were washed and incubated with secondary antibodies at room temperature for 1 h. Protein bands were detected using an Odyssey Infrared Imaging System.

### Statistics

Data are presented as means ± SD of values obtained from at least three independent experiments. The data were plotted using GraphPad Prism (GraphPad Software, Sand Diego, CA), and Image analysis was performed with ImageJ. The student’s t-test was used to test statistical significance between the two groups. For more than two groups, one-way analysis of variance (ANOVA) with SPSS 22.0 software (SPSS Inc.) was performed to analyze differences, as significant differences are indicated, followed by LSD post hoc test. Differences were considered significant at *P < 0.05, **P < 0.01 and ***P < 0.001.

## Results

### Administration of LY450139 Inhibited Ti Particle-Mediated Osteolysis In Vivo

**Fig. 1:**
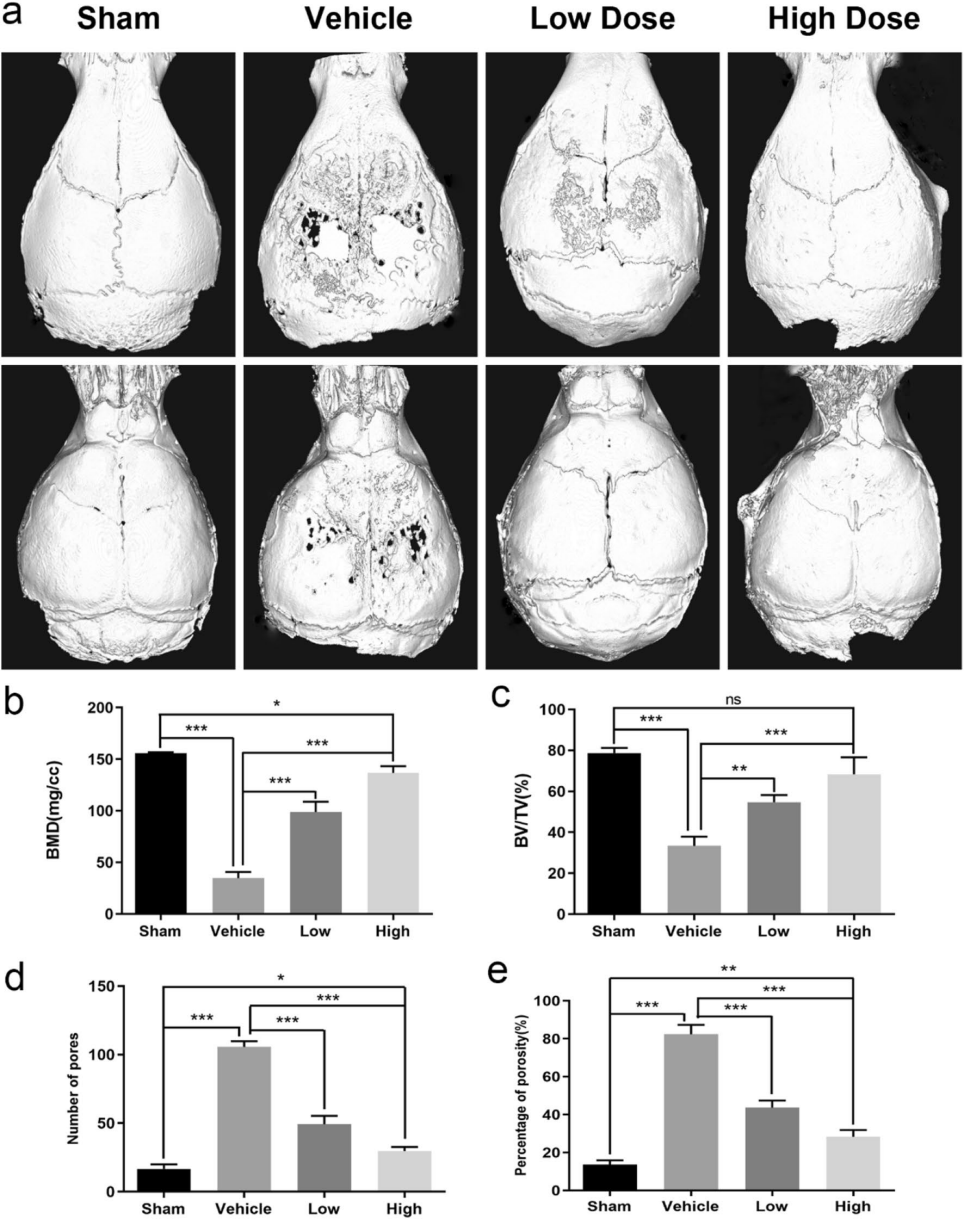
The curative effects of LY450139 on titanium particles stimulated osteolysis animal models in vivo. **a** Representative micro‐CT reconstruction images of murine calvaria from each group (*n* = 5). **b** bone mineral density (BMD). **c** bone volume/total tissue volume (BV/TV). **d** The number of pores and **e** the percentage of porosity within the region of interest (ROI) was measured. (*P < 0.05, **P < 0.01 and ***P < 0.001)

**Fig. 2:**
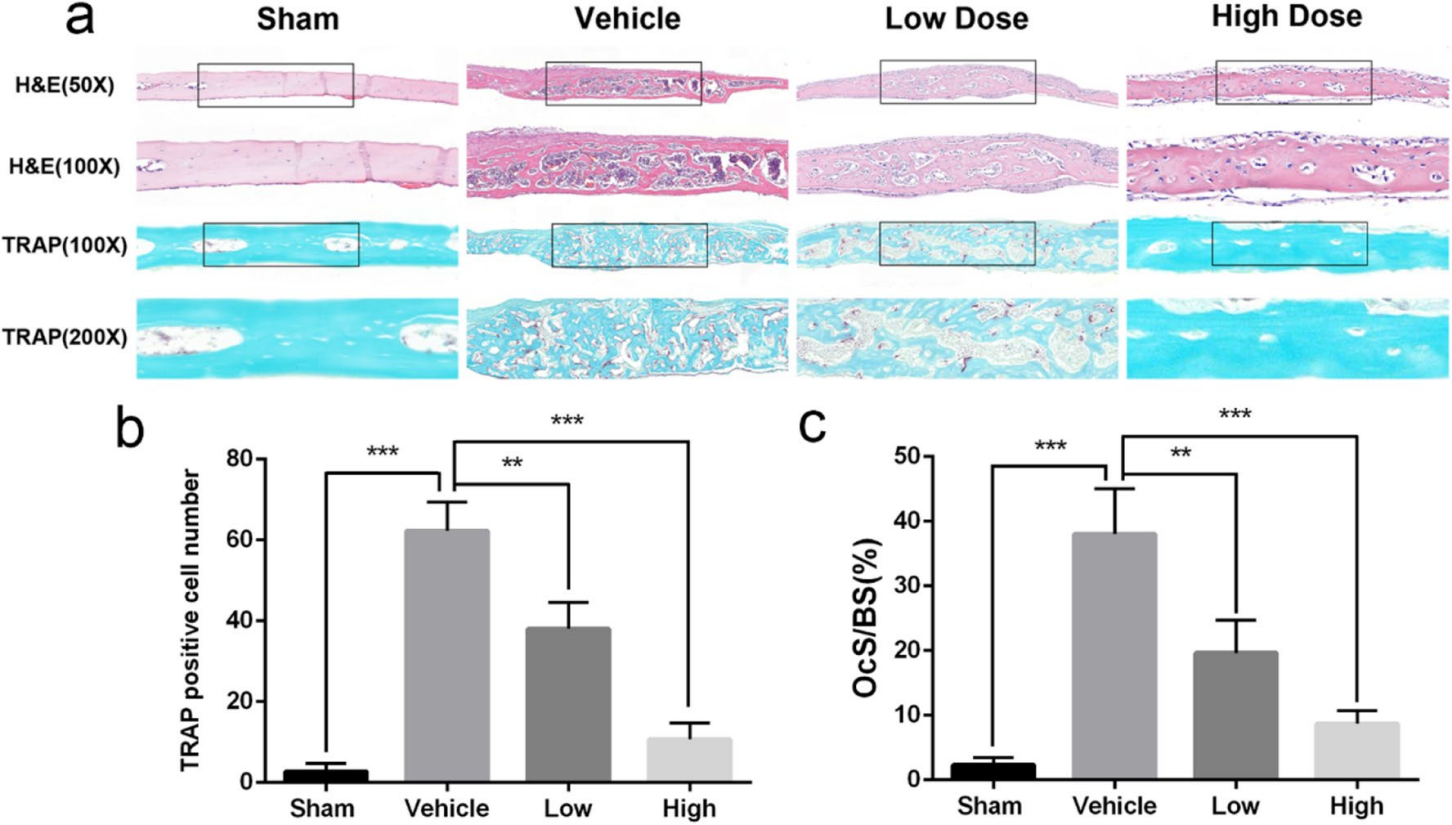
Histological staining of calvaria sections and histomorphometric analysis. **a** Representative hematoxylin and eosin (H&E) staining and TRAP staining. **b** The number of TRAP-positive osteoclasts and **c** the percentage of osteoclast surface per bone surface (OcS/BS, %) was measured in each group (*n* = 5). (*P < 0.05, **P < 0.01 and ***P < 0.001)

Ti particle-induced osteolysis modal was constructed to investigate LY450139 therapeutic effects on osteolysis in vivo. Micro-CT scans showed that embedding titanium particles into the calvaria caused evident bone erosion exhibiting uneven distribution on the bone surface of the vehicle group. In contrast, LY450139 administration significantly attenuated the degree of osteolysis in a dose-dependent manner (Fig. 1a). Quantification of bone parameters showed that compared with the Sham group, the values of BMD and BV/TV significantly decreased while the number of pores and the percentage of porosity significantly increased in the Vehicle group. LY450139 treatment significantly increased BMD, BV/TV, and decreased the number of pores and the percentage of porosity compared to the vehicle group. However, BMD values in the high-dose group were still significantly lower, and the number of pores and the percentage of porosity were significantly higher than Sham group (Fig. 1b–e). Histological analysis further verified that LY450139 attenuated Ti particle-irritated bone resorption in vivo (Fig. 2). Histomorphometric analysis indicated that Ti particles induced more significant osteolytic variation, and TRAP-positive multinucleated cells accumulated more obviously along the eroded bone surface of the vehicle group compared with the sham group. (Fig. 2a). As expected, LY450139 administration reduced the number of osteoclasts and the osteoclast surface/ bone surface (OcS/BS) ratio (Fig. 2b, c). Together, these results revealed that LY450139 administration delayed Ti-mediated bone loss via suppressing osteoclast activity in a dose-dependent manner.

### LY450139 Dose-Dependent Suppressed Osteoclastogenesis Without Cytotoxicity

**Fig. 3:**
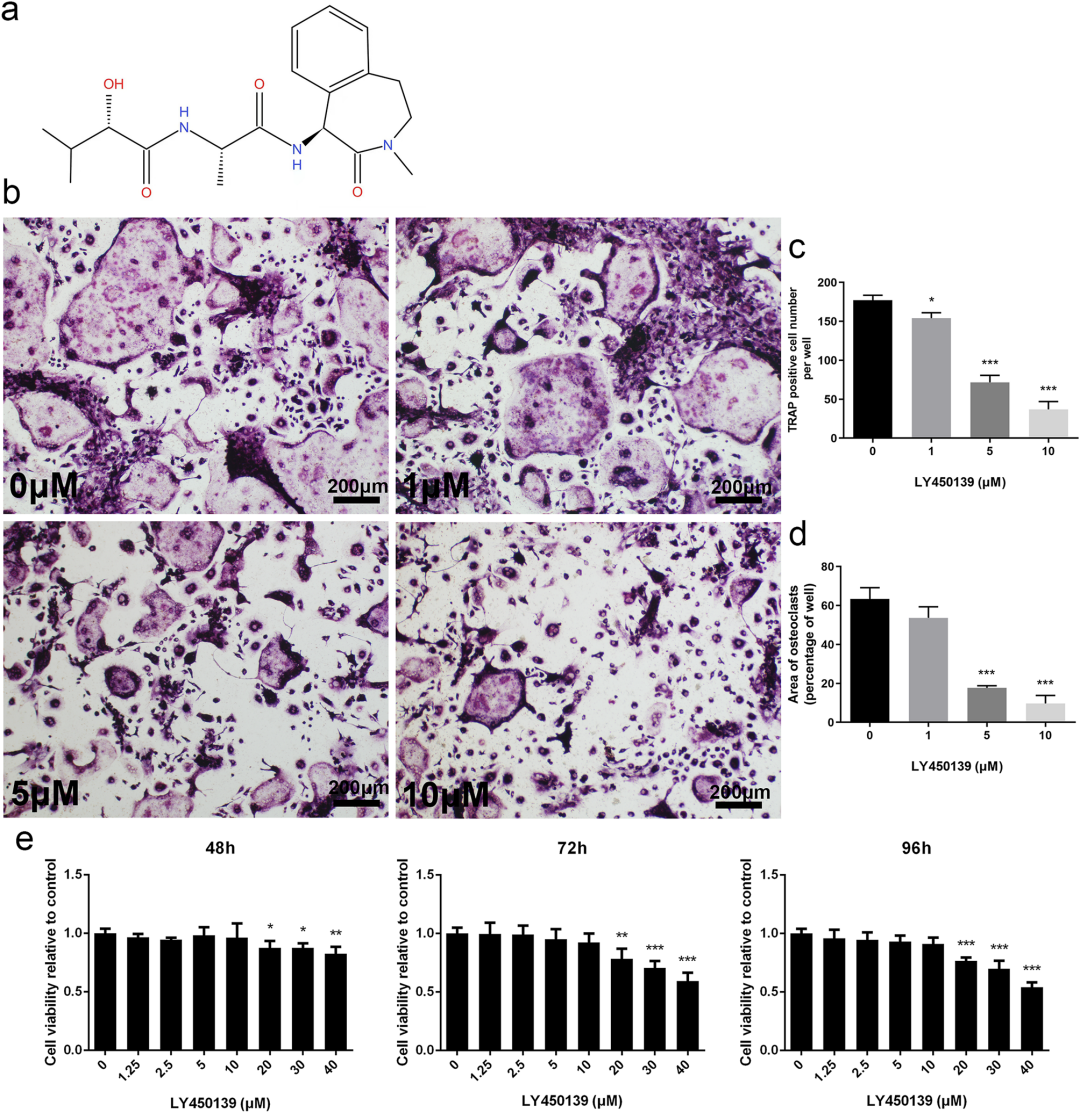
LY450139 inhibited RANKL-induced osteoclast formation in a dose-dependent manner without cytotoxicity. **a** Chemical structure of LY450139: molecular formula C_19_H_27_N_3_O_4_ (97.7% purity) with an average molecular weight of 361.44. **b** BMMs were stimulated with various concentrations of LY450139 (0, 1, 5, 10 μM) in the presence of M-CSF (30 ng/mL) and RANKL (50 ng/mL) for 5 days and then stained with TRAP g. **c, d** The numbers of TRAP-positive multinuclear cells and osteoclast area were measured as described in the methods. **e** BMMs were cultured in 96-well plates and stimulated with M-CSF (30 ng/mL) and RANKL (50 ng/mL) in the presence of the indicated LY450139 concentrations. Cell viability was then measured using the CCK-8 assay at 48, 72, and 96 h. (*P < 0.05, **P < 0.01 and ***P < 0.001)

The chemical structure of LY450139 is shown (Fig. 3a). The effect of LY450139 on RANKL-mediated osteoclast differentiation was validated in vitro. The results showed that numerous TRAP-positive multinucleated osteoclasts were generated in the control group (Fig. 3b). Nevertheless, the area and number of osteoclasts with prominent large pancake contours gradually decreased with increasing doses (1, 5, and 10 uM), demonstrating the inhibitory effects of LY450139 on RANKL-induced osteoclast differentiation in a dose-dependent manner (Fig. 3b–d). To exclude the possibility of reduced osteoclast formation caused by drug cytotoxicity, CCK-8 was used to assess cell viability. As shown in Fig. 3e, LY450139 treatment had no significant cytotoxic effects on BMM precursors at concentrations ≤ 10 uM, the concentration at which LY450139 effectively suppressed osteoclastogenesis. Therefore, the above results suggested that LY450139 attenuated osteoclastogenesis without drug toxicity.

### LY450139 Impairs Osteoclastic Bone Resorption

**Fig. 4:**
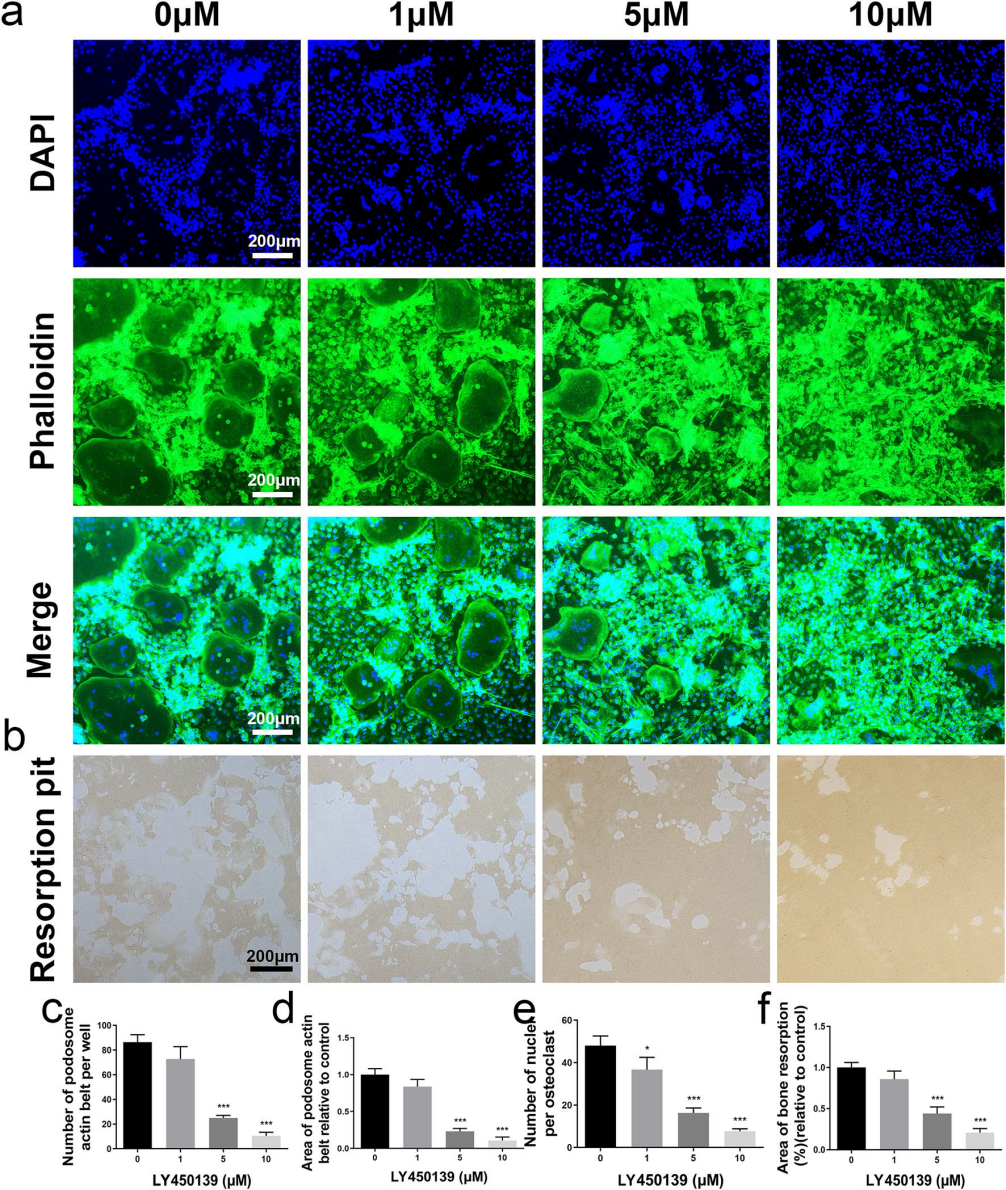
LY450139 inhibited the activity of osteoclast-mediated bone resorption and the formation of podosome actin belts in vitro. **a** Representative confocal images showed the inhibitory effects of LY450139 on osteoclast podosome actin belts. BMMs were stimulated with M-CSF (30 ng/mL), RANKL (50 ng/mL), and various concentrations of LY450139 for 5 days. Osteoclasts were fixed with 4% paraformaldehyde (PFA) and stained with rhodamine‐conjugated phalloidin and DAPI to assess the formation of podosome actin belts. **b** Representative images showed the inhibitory effect of LY450139 on osteoclast-mediated resorption on hydroxyapatite-coated osteo assay plates. **c**–**e** Number and area of podosomal actin belts and number of nuclei per osteoclast. **f** Resorption pit areas in each group were analyzed using Image J software. All experiments were performed at least three times (*P < 0.05, **P < 0.01 and ***P < 0.001)

We used hydroxyapatite-coated plates to indirectly probe the effects of LY450139 on RANKL-stimulated osteoclast bone resorption in vitro. In the RANKL‐the only treated group, mature osteoclasts extensively resorbed hydroxyapatite and left corrosion marks clustered together in large round pancakes (Fig. 4b). In contrast, BMM precursors treated with LY450139 (5 μΜ) effectively inhibited the percentage of osteoclast-mediated hydroxyapatite resorption area by approximately 55%. There was almost no hydroxyapatite plate absorption when osteoclasts were treated with LY450139 (10 μΜ) at higher concentrations (Fig. 4f). Taken together, these data further confirmed that LY450139 was effective in alleviating bone resorption and that LY450139 showed a significant anti-resorptive effect by suppressing osteoclast function.

### LY450139 Suppressed Osteoclastic Podosomal Actin Belt Formation

Osteoclasts from a specialized cell–matrix adhesion structure, the "F‐actin ring", during bone resorption, which defines the bone resorption area. Osteoclasts plated on glass do not form an F‐actin ring but generate a separate molecular structure called the "podosome actin belt" [23]. Therefore, we investigated whether LY450139 could inhibit osteoclastic bone resorption. Results showed that clear structure and the absolute number of podosomal actin belts were detected in the RANKL‐only treated group (Fig. 4a). However, as cells were treated with 5 or 10 μM LY450139, the contour structure of the belts was significantly disturbed as well as the number and area of podosomal actin belts were decreased dose-dependently (Fig. 4c–e). Taken together, these findings indicated that LY450139 treatment suppressed podosomal actin belt formation and osteoclast fusion in vitro.

### LY450139 Suppressed Expression of Osteoclast‐Specific Genes

**Fig. 5:**
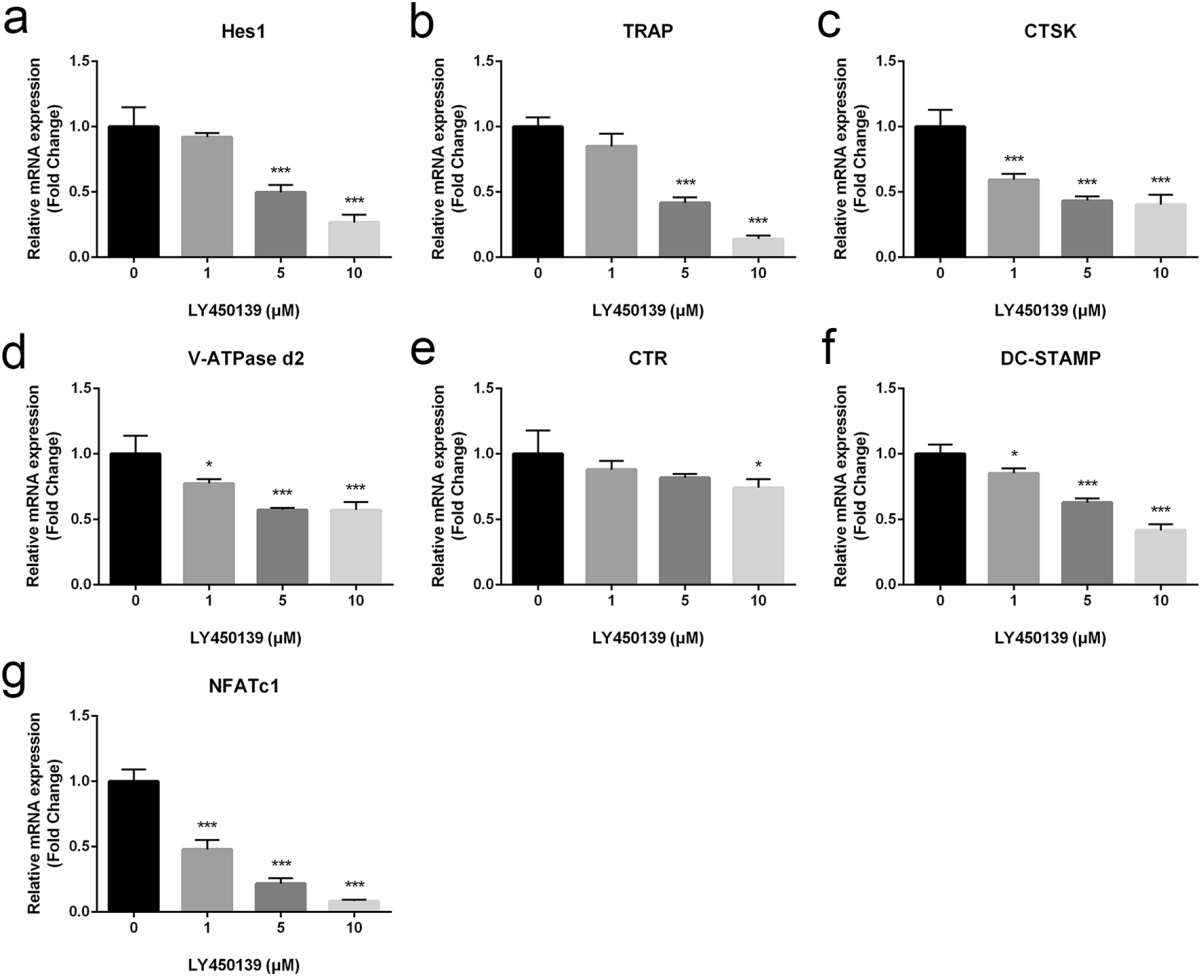
LY450139 downregulated expression levels of RANKL-induced osteoclast-specific genes. BMMs were stimulated with M-CSF (30 ng/mL), RANKL (50 ng/mL), and various concentrations of LY450139 for 5 days. **a**–**g** The expression levels of osteoclast-specific marker genes, including Hes1, TRAP, CTSK, V-ATPase d2, CTR, DC-STAMP, NFATc1, were analyzed by real-time PCR. All experiments were performed at least three times. (*P < 0.05, **P < 0.01 and ***P < 0.001)

It is well known that the upregulation of specific marker genes induces the osteoclastic formation and bone resorption [24]. To further verify the antagonistic effect of LY450139 on osteoclast formation, we assayed mRNA levels by qRT-PCR to analyze the expression of osteoclast-specific genes. Our data showed that osteoclast-specific genes such as TRAP, CTSK, V-ATPase d2, CTR, DC-STAMP, NFATc1, and downstream target gene Hes1 of Notch pathway all showed significant expression under RANKL stimulation. However, the expression of these genes was markedly downregulated in the presence of LY450139, in a dose-dependent manner (Fig. 5a–g). Collectively, these results further confirmed that LY450139 attenuates the expression of osteoclast-specific genes during osteoclastogenesis in vitro.

### LY450139 Depressed Osteoclastogenesis by Inhibiting NF‐κB and Notch Signaling

**Fig. 6:**
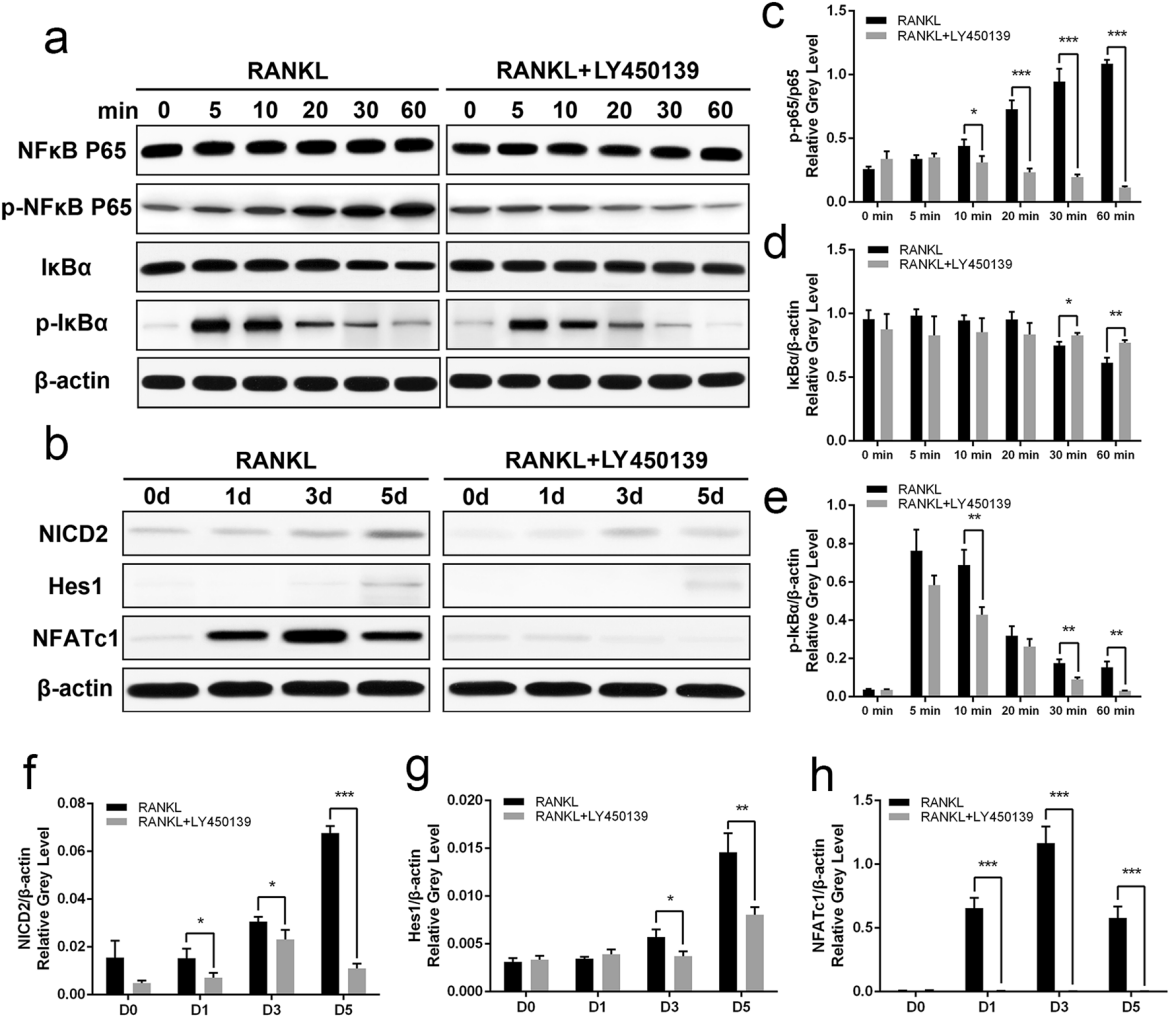
LY450139 inhibited RANKL-induced NF-κB and Notch signaling pathways. **a** LY450139 inhibited RANKL‐induced activation of the NF-κB pathway. BMMs were pretreated with or without LY450139 (10 μM) for 2 h and then stimulated with RANKL (50 ng/mL) for the indicated time points. Cells were lysed, and the extracts were analyzed by western blotting using the indicated antibodies. **b** LY450139 prevented the cleavage of Notch2 and the expression of downstream related target proteins. BMMs were stimulated with M-CSF (30 ng/mL) and RANKL (50 ng/mL) in the presence or absence of LY450139 (10 μM) for 0, 1, 3, or 5 days. The extracted lysates were analyzed by western blotting with antibodies against Notch2, hes1, NFATc1, and β-actin. **c**–**e** Quantitative densitometric analysis of IκBa, p-IκBa, NFκB p65, and p-NFκB p65 expression was analyzed and normalized to β-actin using Image J v6.0 software. **f**–**h** Quantitative densitometric analysis of Notch2, hes1, NFATc1 expression was analyzed and normalized to β-actin using Image J v6.0 software. All experiments were performed at least three times. (*P < 0.05, **P < 0.01 and ***P < 0.001)

To understand the underlying molecular mechanisms of LY450139 and the critical roles of signaling pathways and related transcription factors in osteoclastogenesis and differentiation, we screened the signaling pathways and key molecules associated with osteoclast formation. Our western blotting assays demonstrated that both activation and translocation of NF-κB were markedly inhibited in the LY450139 (10 μM)-treated group compared to the control group (Fig. 6a). With RANKL treatment, IκBα degradation was observed at 30, and 60 min. However, when BMMs were pretreated with LY450139 (10 μM) before RANKL stimulation, the degradation levels were markedly attenuated. p-NF‐κB p65 was also attenuated at 10,20, 30 min, and 60 min. Notch signaling pathway also plays an essential role in the regulation of bone formation. Our results showed that LY450139 had a suppressive effect on the expression of Notch2 in LY450139 (10 μM)-treated groups, compared with the control group at 1 day, 3 day, and 5 day (Fig. 6b). Notch2 has undergone nuclear translocation and plays a role in mediating downstream target genes. Hes1 and NFATc1 are essential transcription factors in mediating the expression of downstream‐related genes. We found that LY450139 reduced the protein levels of Hes1 and NFATc1 after 3 days and 1 day. Taken together, our results confirm that, after RANKL induction, LY450139 attenuates the activity of NF‐κB and Notch pathways, as well as the expression of downstream transcription factors (Hes1 and NFATc1).

## Discussion

Total hip replacement (THR) has been widely regarded as one of the most clinically successful surgical procedures for ameliorating pain, improving function and mobility in patients with end‐stage joint disease. Unfortunately, few hip prostheses can survive more than 25 years. The most common cause of failure in joint replacement surgery is aseptic loosening [25]. At present, aseptic loosening is caused mainly by osteoclast activation induced by wear debris from the hip joint prosthesis [5, 26]. Therefore, targeted inhibition of key signaling pathways involved in osteoclast differentiation may ameliorate bone mass loss, decelerate degeneration of the trabecular bone, and increase bone mineral density recovery, indicating a promising new therapeutic approach for addressing wear-debris-induced osteolysis. Semagacestat (LY450139) is developed as a potential treatment for Alzheimer ‘s disease, which blocks the amyloid β-protein (Aβ) production, reduces the pathological process of Alzheimer's disease to a certain extent. Unfortunately, trials for phase 3 trials in humans with Alzheimer’s disease had been unsuccessful, because the results of the trial showed that it did not improve the cognitive status, and caused a significant worsening ability of daily living [27]. Additionally, there were more adverse events including alterations of immune cells, gastrointestinal symptoms, infections, skin reactions, and skin cancers [27, 28], which have been added to the limitations in this study. Despite adverse events and lack of efficacy limit the utility potential of agents. However, the vital role that γ-secretase plays as a potential intervention point targeting dysregulated Notch signaling still has attracted increasing interest. Previous studies reported that γ-secretase activity had also been associated with breast cancer cells and melanoma cells [29, 30], myeloma [31], cardiomyopathy [32], and kidney and immune disorders [33, 34]. Therefore, further research on the functional roles of γ-secretase may provide novel insight for the targeted therapy of these diseases. Whether LY450139 could inhibit osteoclast formation and function is still unknown. Our study result confirmed that LY450139 serve as an effective candidate compound for the treatment and/or prevention of Ti particle-induced pathological bone loss in a murine model.

In this study, our results showed that LY450139 could ameliorate osteolysis in the Ti-particle-induced mice calvarial osteolysis model. Micro-CT data analyses demonstrated that osteolysis in response to titanium achieved improvement following LY450139 administration, accompanied by the increase of BMD and BT/TV in a dose-dependent manner, in addition to a marked reduction of TRAP-positive multinucleated cells number in calvaria sections. This observation is consistent with published reports describing restraint osteolysis activity of γ-secretase inhibitor [35–37]. Interestingly, we also found that the BMD value in the high-dose group did not fully regained the normal level, attributable to restraining an individual signaling pathway is incomplete effectiveness on blocking osteoclast activity regulated by the multiple cellular signaling pathways. Another reason is that Titanium particles stimulating response inhibits the differentiation of mesenchymal stem cells and mineralization of osteoblasts, which then leads to the increase of bone loss [38–40]. Hence, LY450139 delay the occurrence and progression of osteolysis rather than completely blocking osteolysis and restoring bone mass.

Further studies were performed to explore the possible molecular mechanism of the inhibitory effects. We found that the mRNA expression level of osteoclast-related genes decreased following the treatment with LY450139. Several previous studies reported that RANKL binding to RANK activates several vital signaling pathways in osteoclast progenitors, such as the MAPK [41], JNK [42], and NF‐κB signaling pathways [43]. In this study, we demonstrated that LY450139 inhibited the activation of NF-kB, which involved nuclear translocation and phosphorylation of p65 and the phosphorylation and degradation of IkBa, as determined by western blotting. Furthermore, LY450139 reduced the expression of downstream osteoclastogenic transcription factors including c-Fos and NFATc1 and subsequently inhibited the expression of NFATc1 regulated osteoclastic marker genes including CTSK, TRAP, CTR, V-ATPase d2, and Dc-stamp.

Notch signaling pathway plays a critical role in osteoclast/osteoblast differentiation and function, bone homeostasis, and bone remodeling [18]. Notch complexes are transmembrane receptors composed of four Notch receptors (Notch1-4) [44]. Preliminary research shows that four different types of receptors play different roles in the generation of osteoclasts. Notch1 inhibits osteoclast precursors and suppresses osteoclastogenesis by direct and indirect mechanisms [45]. The genetic deletion of Notch1 in osteoclast precursors enhances osteoclastogenesis, whereas the overexpression of Notch1 suppresses NFATc1 transcription and osteoclast differentiation [45, 46]. Notch2 induces osteoclast formation, thus showing an opposite effect to that of Notch1 [47]. The results of the western blot revealed that LY450139 significantly suppressed RANKL-induced Hes1 and NFATc1 expression at the protein level. Previous studies have shown a complex context-dependent cross-talk between Notch and NF-κB signaling in osteoclastogenesis [47, 48]. Interactions of Notch2 with the p65 subunit of NF-κB in osteoclast precursors lead to the transcription of NFATc1 [49]. The levels of Hes1 increase during osteoclast differentiation in parallel to those of Notch2 [49, 50]. In contrast, LY450139 significantly decreased the expression of NFATc1 by blocking Notch cleavage and liberation of NICD2, thereby, the downstream product Hes1 was reduced. NICD2 of Notch2 in the Notch pathway interacts with nuclear factor NF-κB to upregulate c-Fos and NFATc1 transcription and to promote osteoclast generation. With the intervention of γ-secretase inhibitor or Notch2 mRNA inhibitor, RANKL-mediated osteoclast formation and function was weakened. These results further confirmed that LY450139 disturbs RANKL-mediated osteoclastogenesis and functions via regulation of osteoclast marker gene expression in Notch and NF-κB signaling pathways.

Notwithstanding these insights, there are several limitations in this study. First, clinical peri-prosthetic osteolysis is partially induced by polyethylene rather than Ti particles, which is a complex process relying on numerous cell types and signals. It has been documented that both titanium and polyethylene could activate osteoclast differentiation and trigger noticeable osteolytic effects in vivo. Second, osteolysis model of murine calvarias may not be optimal for interpretation of clinical peri-prosthetic osteolysis. As noted by von Knoch et al. [51], wear particles are continuously released at lower concentrations from the surface of the implant, rather than t injection at one-time point. The administration of only 2 weeks is very short-lived in the clinical practice. Third, there are several reports of LY450139 (semagacestat) as a potential treatment for Alzheimer's disease [29, 52]. However, some patients in a phase 3 trial receiving LY450139 showed immense side effects, such as skin cancer and infections [19, 27]. Hence, the side effect of LY450139 is another issue we should consider. It is unclear whether those doses and dosing intervals of LY450139 resulted in optimal peak therapeutic levels or maintained an optimal therapeutic blood concentration, and what adverse reactions can be produced by long-term administration. Such problems are expected to be answered in future research.

Taken together, our studies indicated that LY450139 had inhibitory effects on RANKL-mediated osteoclastogenesis in vitro and Ti particle-induced inflammatory osteolysis in vivo. Furthermore, these inhibitory effects were accomplished in part by attenuation of Notch/HES1/NF-κB‐mediated NFATc1 induction. Therefore, our findings suggested that LY450139 had the therapeutic potential for preventing or treating peri-prosthetic osteolysis and other osteolytic diseases.

## Data Availability

The data that support the findings of this study are available from the corresponding author or the first author upon reasonable request.
